# Multiple Modes of Action of the Squamocin in the Midgut Cells of *Aedes aegypti* Larvae

**DOI:** 10.1371/journal.pone.0160928

**Published:** 2016-08-17

**Authors:** Marilza da Silva Costa, Sérgio Oliveira de Paula, Gustavo Ferreira Martins, José Cola Zanuncio, Antônio Euzébio Goulart Santana, José Eduardo Serrão

**Affiliations:** 1 Department of Entomology, Federal University of Viçosa, Viçosa, Minas Gerais, Brazil; 2 Department of General Biology, Federal University of Viçosa, Viçosa, Minas Gerais, Brazil; 3 Institute of Chemistry and Biotechnology, Federal University of Alagoas, Maceió, Alagoas, Brazil; Instituto Nacional de Salud Pública, MEXICO

## Abstract

Annonaceous acetogenins are botanical compounds with good potential for use as insecticides. In the vector, *Aedes aegypti* (L.) (Diptera: Culicidae), squamocin (acetogenin) has been reported to be a larvicide and cytotoxic, but the modes of action of this molecule are still poorly understood. This study evaluated the changes in the cell morphology, and in the expression of genes, for autophagy (*Atg1* and *Atg8*), for membrane ion transporter *V-ATPase*, and for water channel aquaporin-4 (*Aqp4*) in the midgut of *A*. *aegypti* larvae exposed to squamocin from *Annona mucosa* Jacq. (Annonaceae). Squamocin showed cytotoxic action with changes in the midgut epithelium and digestive cells of *A*. *aegypti* larvae, increase in the expression for autophagy gene *Atg1* and *Atg8*, decrease in the expression of *V-ATPase*, decrease in the expression of *Aqp4* gene in LC_20_ and inhibition of *Apq4* genes in the midgut of this vector in LC_50_. These multiple modes of action for squamocin are described for the first time in insects, and they are important because different sites of action of squamocin from *A*. *mucosa* may reduce the possibility of resistance of *A*. *aegypti* to this molecule.

## Introduction

Some natural products from plants has been identified and isolated with insecticidal against various insects. These have been obtained from plants and have been identified and isolated (for review see Okwute) [1]. However, the focus of these studies is to find molecules with potential for the synthesis of new insecticides, which act against several molecular targets in the insects [2].

Among these natural compounds, acetogenins from Annonaceae plants are compounds with good potential for use as insecticides [3]. It is quite likely that there are other botanical compounds whose modes of action of acetogenins against insects are poorly understood. In mammals, acetogenins are potent inhibitors of mitochondrial complex I, inhibiting nicotinamide adenine dinucleotide hydride (NADH) ubiquinone oxidoreductase, an essential enzyme in the complex I [4]. Acetogenins bind on the catalytic site of ubiquinone in the complex I as well as in the microbial glucose dehydrogenase inhibiting NADH oxidase in cancer cells [4].

Because acetogenins affect cellular respiration, especially during the conversion of energy [5], they may be important components in management programs to avoid insect resistance to these molecules. Molecules with specific or multiple sites of action may be a power tool in mitigating insect resistance to insecticides. In addition, unexplored compounds may have different sites of action, may be selective in safety to humans and the environment [6]. In general, secondary plant metabolites play some role of interference in critical components of the cell signaling system, nervous system (e.g., neurotransmitter synthesis, receptors activation and signal transduction), metabolic pathways and the enzymes activities [7].

The search for alternatives to control disease vectors, especially *Aedes aegypti* (Diptera: Culicidae), the main vector for yellow fever, dengue [8], chikengunya fever [9] and zika virus [10] is urgently required. This mosquito is difficult to control because it adapts well to the environment due to its resilience and ability to overcome population disturbance caused by human interventions [11,12]. In addition, *A*. *aegypti* has developed rapid resistance for conventional insecticides, and to date, its resistance is reported for 35 active ingredients [13]. In Brazil, the main strategies used in the control of *A*. *aegypti* are based on the chemicals that act as acetylcholinesterase inhibitors, axonic nerve poisons, and insect growth regulators [14,15]. Thus, the use of alternative molecules with multiple sites of action is important to control this resistant species of mosquito.

In *A*. *aegypti*, different classes of acetogenins have been tested [16–18], and squamocin (acetogenin from *A*. *squamosa* seed) had cytotoxic and larvicidal activities against this vector [18]. However, as with many other botanical molecules, there are no data about its modes of action in this insect. In this study, we tested a squamocin isolated from *A*. *mucosa* seeds against the larvae of *A*. *aegypti*, and describe the changes in the cell morphology, and in the expression of genes involved in cell death processes and in plasma membrane transport in the midgut, thus increasing our understanding of the possible modes of action of this molecule.

## Materials and Methods

### Acetogenin

The squamocin was obtained from the Research Laboratory of Natural Resources (LPqRN) of the Federal University of Alagoas, Maceió, Alagoas, Brazil. Squamocin (CAS number: 120298-30-8) is a white solid wax obtained from a methanolic extraction of *Annona mucosa* seeds following by a successive partition with chloroform (85.4 g; 57.3%). This compound was pre-solubilized in 1% dimethylsulfoxide and dissolved in distilled water, resulting in a stock solution of 10 μg/mL.

### Insects

Third instar larvae of *A*. *aegypti* previously fed with cat food (Whiskas) were obtained from mass rearing in the insectary of the Laboratory of Molecular Biology of Insects of the Federal University of Viçosa (UFV), Minas Gerais, Brazil. The insect colonies and the bioassays were performed at 25 ± 2°C, with a 12 hours photoperiod.

Preliminary tests were conducted to determine the toxicity of squamocin, and to set its lethal concentration (LC) and lethal time (LT) against third instar larvae of *A*. *aegypti*, and the bioassays were performed with the sublethal concentrations LC_20_ and LC_50_, because higher concentrations these larvae have a high level of lethargy.

### Midgut cytotoxicity

The *A*. *aegypti* larvae were exposed to the acetogenin at concentrations of 0.004 μL/mL (LC_20_) and 0.01 μL/mL (LC_50_) for 6 and 12 hours, along with control with untreated larvae at the same times. For analysis in light microscope, ten larvae per treatment were dissected in 2% paraformaldehyde fixative solution and stored for 12 hours at 4°C. Then, the samples were dehydrated in a graded ethanol series (30–100%) and embedded in historesin (JB4 Polysciences). Slices of 5 μm thickness were stained with hematoxyline and eosin and, examined and photographed in light microscope (Olympus BX60).

For ultrastructural analyses of the midgut in *A*. *aegypti* larvae treated with acetogenin at LC_20_ and LC_50_ for 6 and 12 hours, the organs were transferred to 2.5% glutaraldehyde in 0.1 M sodium cacodylate, pH 7.2 for 2 hours. The samples were post-fixed in 1% osmium tetroxide for 2 hours at room temperature in the dark, dehydrated in a graded of ethanol series and embedded in LR White resin [19]. Ultrathin sections were stained with 2% aqueous uranyl acetate and 1% lead citrate and examined in a Zeiss EM 109 or LIBRA 120 transmission electron microscope.

### RNA preparation

The midgut from 10 larvae exposed to each squamocin concentration of 0.004μL/mL (LC_20_) and 0.01μL/mL (LC_50_) for 0.5, 1, 2, 3 and 4 hours for *Atg1* and *Aqp4* and 5, 10, 15 and 17 hours for *Atg8* and *V-ATPase*, and the control, were dissected and transferred to RNAlater (Sigma-Aldrich). Then the samples were transferred to 500 μL of Tri-reagent (Sigma), homogenized and centrifuged at 12,000 × *g* for 10 minutes at 4°C. To the supernatant was added 100 μL of chloroform, following incubation for 10 minutes and centrifugation at 12,000 × *g* for 15 minutes at 4°C. The aqueous phase was transferred to 250 μL of 2-propanol, incubated for 10 minutes in ice and centrifuged at 12,000 × *g* for 10 minutes. The pellet was washed twice with 500 μL of 75% ethanol and centrifuged at 12,000 × *g* for 5 minutes. The pellet was then air dried and resuspended in 20 μL of ultrapure water. The amount of RNA was determined with a NanoDrop Lite Spectrophotometer (Thermo Scientific), and its sample integrity was verified by agarose gel electrophoresis in Tris/Borate/EDTA buffer.

### Synthesis of cDNA

The RNA (500 ng) obtained from the midgut of *A*. *aegypti* larvae treated with squamocin at each concentration and time and control was transferred to 1 μL of 2.5 mM dNTP mix (dATP, dGTP, dCTP and dTTP), 1 μL 100 μM primers oligo (dT) and ultrapure water to 10 μL final volume. After mild vortexing, the samples were incubated for 3 minutes at 70°C and cooled in ice. Next, were added 4 μL of buffer (500 mM Tris-HCl pH 8.3, 500 mM KCl, 30 mM MgCl_2_, 50 mM DTT), 1 μL of M-MuLV reverse transcriptase enzyme (Invitrogen) and ultrapure water to 20 μL final volume. These samples were incubated for 1 hour at 37°C, following enzyme inactivation at 72°C for 15 minutes. The cDNA obtained was quantified in a spectrophotometer NanoDrop Lite.

### *Real Time qPCR* (RT-qPCR)

The genes tested were *Atg1* and *Atg8* for autophagy, *Aqp4* for aquaporin, *V-ATPase* type for membrane ion transport, and the *rp7S* ribosomal protein as reference from their primers (Table 1) [20].

**Table 1:** Primers used in the bioassay of gene expression by real-time quantitative PCR.

Gene	Forward primer	Reverse primer
*Atg1*	5’CCTGACTGGTAAGGCACCAT 3’	5’GTTGTTGCTGCTGGAGTTGA3’
*Atg8*	5’GGAAGAACACCCATTCGAGA3’	5’AGCCGATGTGGTGGAAT3’
*V-ATPase*	5’GTTGTTCTGGCTCTGCTGTTA3’	5’GAGTGTTCTCGATAAGCCATAATC 3’
*Aqp4*	5’ATGCCACTGCTTGTCCCTAC 3’	5’TTTCCGAAATGACCTTGGAG 3’
*rp7S*	5’TCAGTGTACAAGAAGCTGACCGGA 3’	5’TTCCGCGCGCGCTCACTTATTAGATT 3’

For the determination of gene expression, cDNA samples from the midgut of *A*. *aegypti* larvae treated at different concentrations and times of acetogenin and control were submitted to RT-qPCR (Eco Real-Time PCR System- Illumina) in quadruplicate using the quantitation fluorescence kit GoTaq® Master Mix (Promega). The final primer concentration was 0.1 μM.

The relative expression of the genes was obtained using the Cycle Threshold method. The Ct values were subjected to 2^-ΔΔCt^ to determine the gene expression [21].

### Statistical analysis

The gene expression data for *Atg1*, *Atg8*, *Aqp4* and *V-ATPase* were subjected to one-way analysis of variance, considering as factors the lethal concentration, and the exposition time followed by a post-hoc Tukey HSD test at 5% significance level.

## Results

Squamocin at sublethal concentrations LC_20_ and LC_50_ showed cytotoxic effect in the midgut cells of *A*. *aegypti* larvae exposed for 6 and 12 hours, although these changes were not dose dependent.

**Fig 1:**
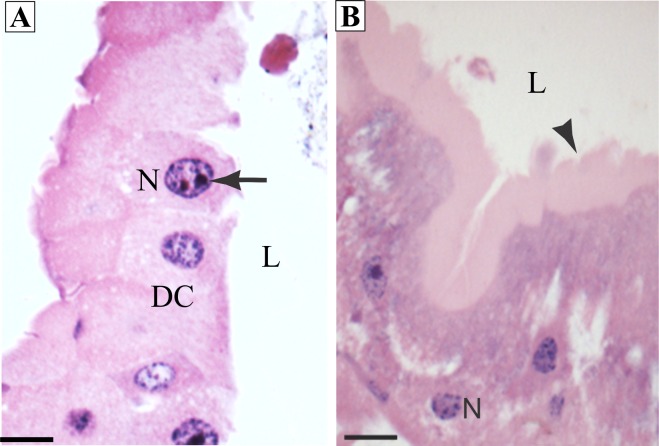
Photomicrographs of the midgut of *Aedes aegypti* third instar larvae (control). (A) Epithelium with a single layer of columnar digestive cells (DC), with spherical nucleus (N) containing nucleolus (arrow). (B) Epithelium showing well-developed apical brush border (arrowhead). L = lumen. Bar = 5μm.

In the control insects, the midgut digestive cells were columnar, with homogenous cytoplasm, median spherical nucleus with nucleoli (Fig 1A), and with a well-developed apical brush border (Fig 1B).

**Fig 2:**
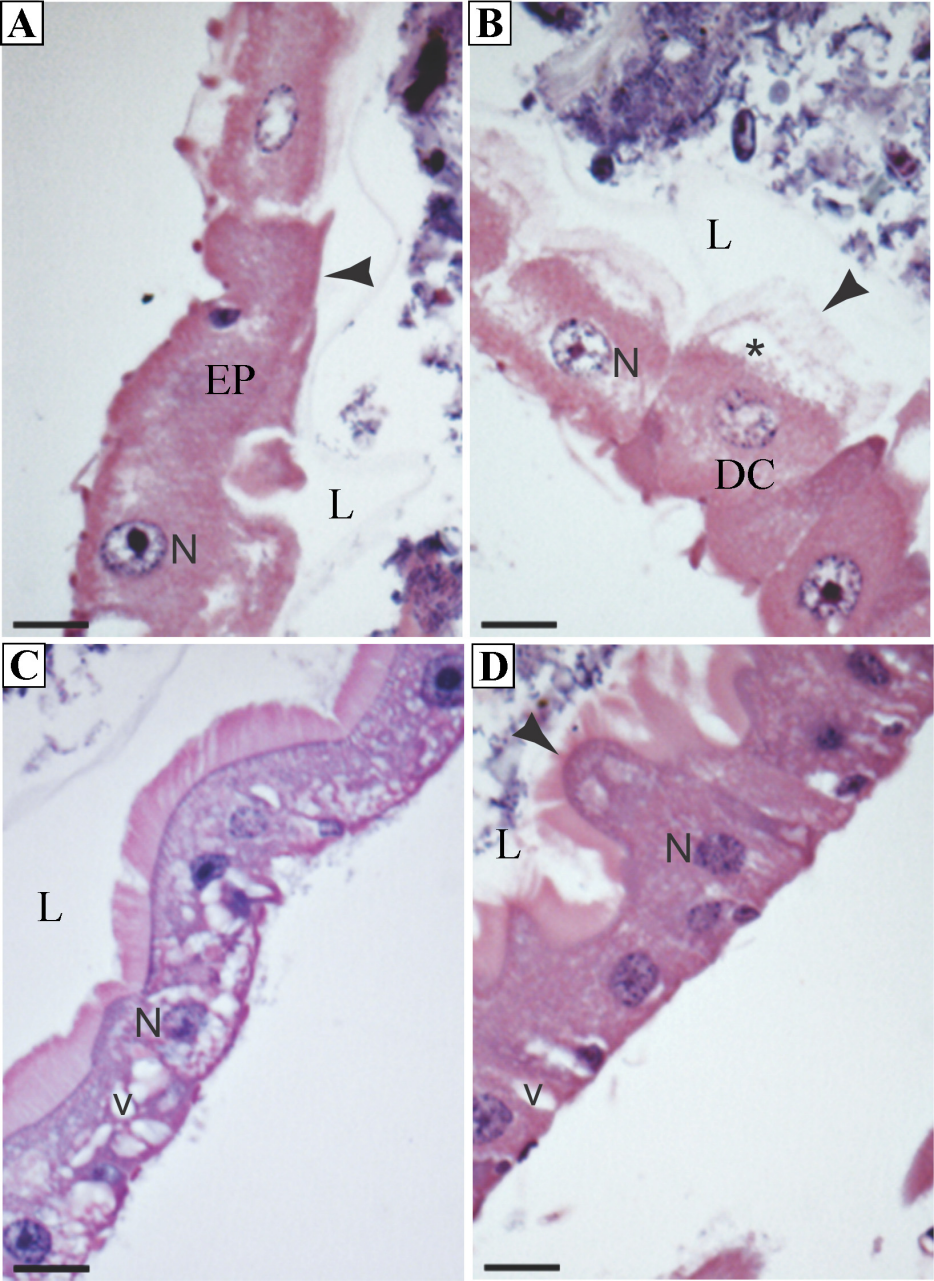
Photomicrographs of the midgut in *Aedes aegypti* third instar larvae exposed to sublethal doses LC_20_ and LC_50_ of squamocin. (A) Disorganized midgut epithelium (EP) (LC_20_). (B) Digestive cells (DC) with vacuoles (*) in the apical cytoplasm and disorganized brush border (arrowhead) (LC_50_). (C) Digestive cells with vacuoles (v) in the basal cytoplasm (LC_20_). (D) Digestive cells with damaged striated border (arrowhead) and vacuoles (v) (LC_20_). N = nucleus, L = lumen. Bars = 5μm.

All larvae exposed to squamocin, independently of doses and exposure times, showed damaged digestive cells (Fig 2A), with presence of vacuoles in the apical and basal cytoplasm (Fig 2B and 2C), and disorganized brush border (Fig 2B and 2D).

**Fig 3:**
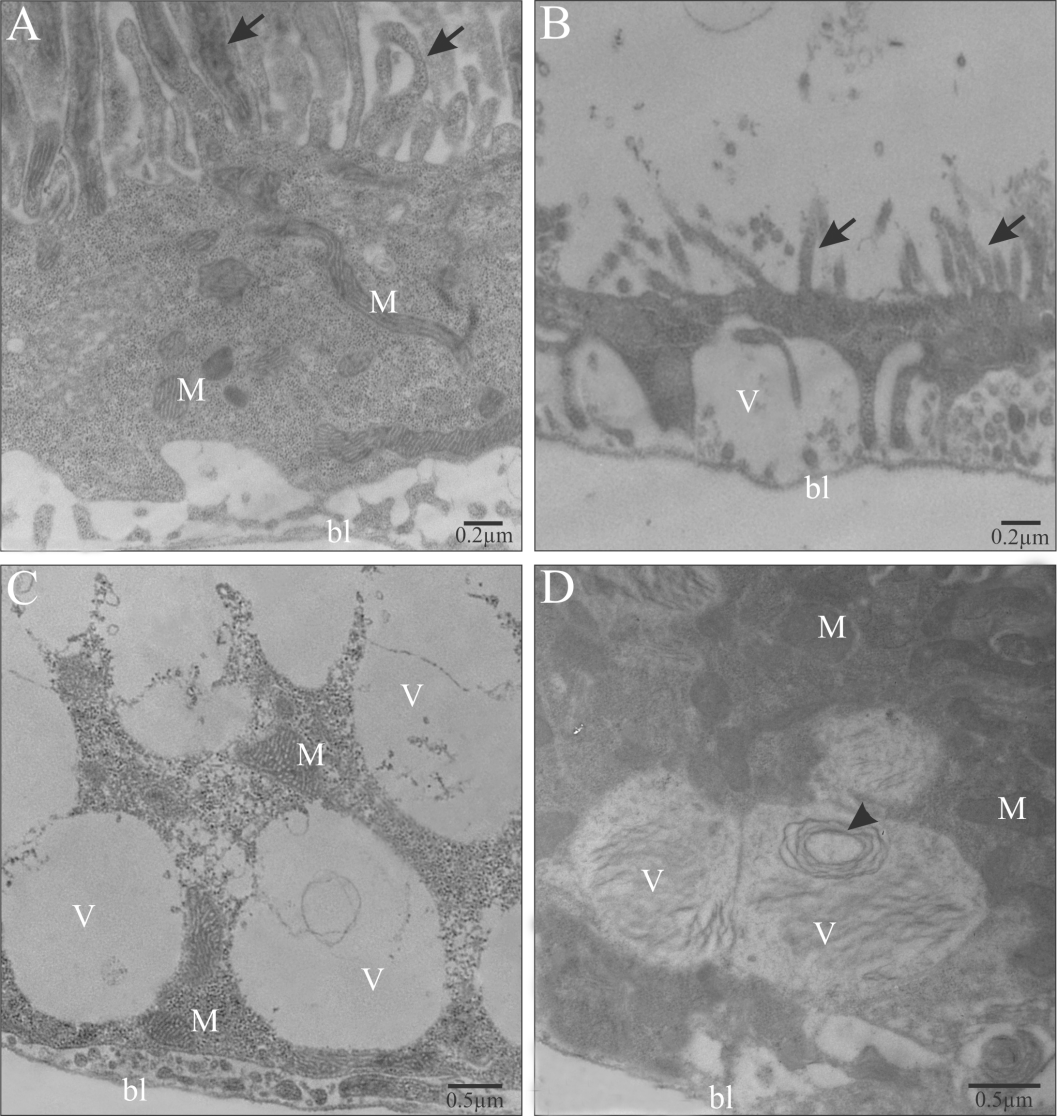
Transmission electron micrographs of the digestive cells in the midgut of *Aedes aegypti* third instar larvae. (A) Midgut cell with long microvilli (arrows) associated with mitochondria (M) in the control larvae. (B) Midgut cell showing disorganized microvilli (arrows) in larvae exposed to LC_50_ acetogenin. (C) Middle-basal cell region showing mitochondria (M) and large vacuoles (V) in the larvae exposed to LC_20_ acetogenin. (D) Middle-basal cell region showing presence of large vacuoles (V) with lamellar content (arrowhead) in larvae exposed to LC_20_ acetogenin. bl–basal lamina.

Ultrastructural analyses of the midgut cells in the *A*. *aegypti* larvae showed that control ones had digestive cells with well-developed mitochondria and microvilli (Fig 3A), whereas those exposed to LC_20_ and LC_50_, independently of exposure times, showed damages in the cell surface with microvilli loses (Fig 3B) and cytoplasm containing many large vacuoles with lamellar content (Fig 3C and 3D).

To verify whether morphological changes in the digestive cells of *A*. *aegypti* larvae exposed to sublethal concentrations of squamocin results from the action of this molecule in cell death processes and/or membrane transporters, some gene expressions were evaluated.

**Fig 4:**
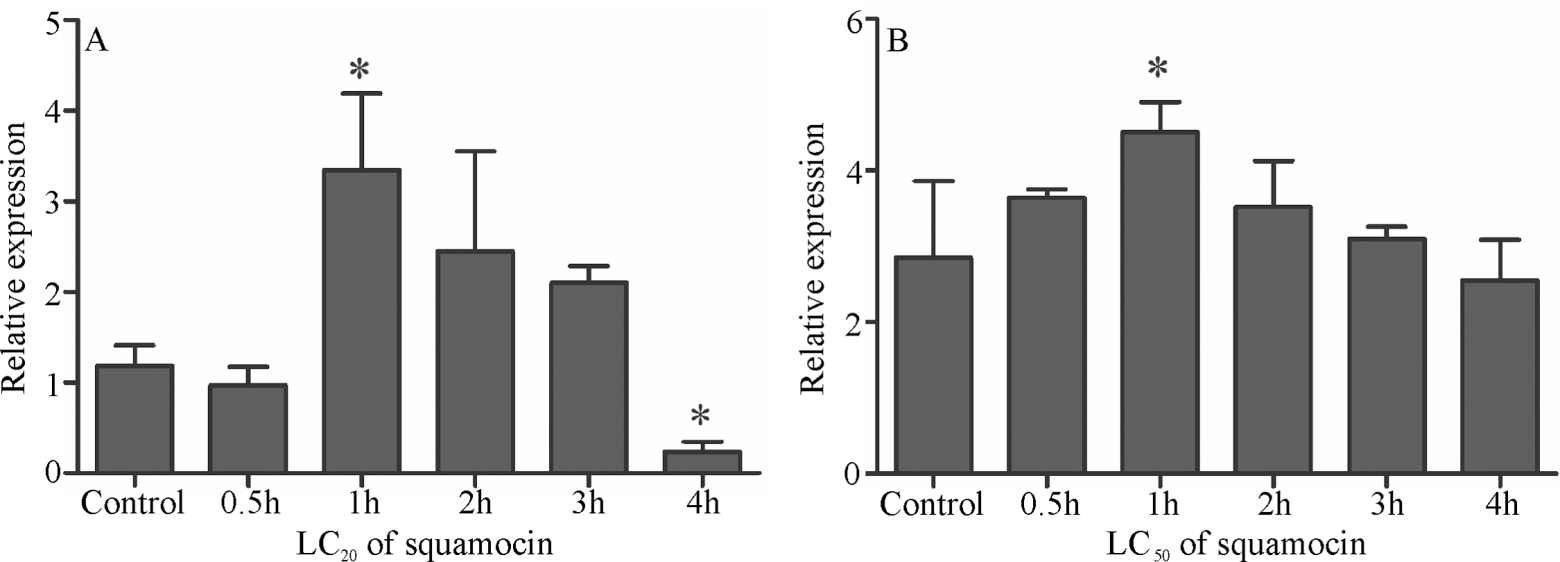
Relative mRNA levels of *Atg1* in the midgut of *Aedes aegypti* third instar larvae exposed to sublethal doses of squamocin and at different times. (A) Lethal concentration of 20% of population (LC_20_). (B) Lethal concentration of 50% of population (LC_50_). The *y-axis* indicate the relative gene expression, corresponding to the *Atg1* mRNA levels relative to ribosomal protein rp7S (reference) gene mRNA level (mean ± se). * *p* < 0.05.

The *Atg1* expression in the midgut of *A*. *aegypti* larvae in LC_20_ was higher after 1 hour of exposure to squamocin when compared to the other times and the control (F_5,12_ = 11.16; p = 0.0004) (Fig 4A) in the same manner as in LC_50_ (F_5,12_ = 4.571; p = 0.0145) (Fig 4B).

**Fig 5:**
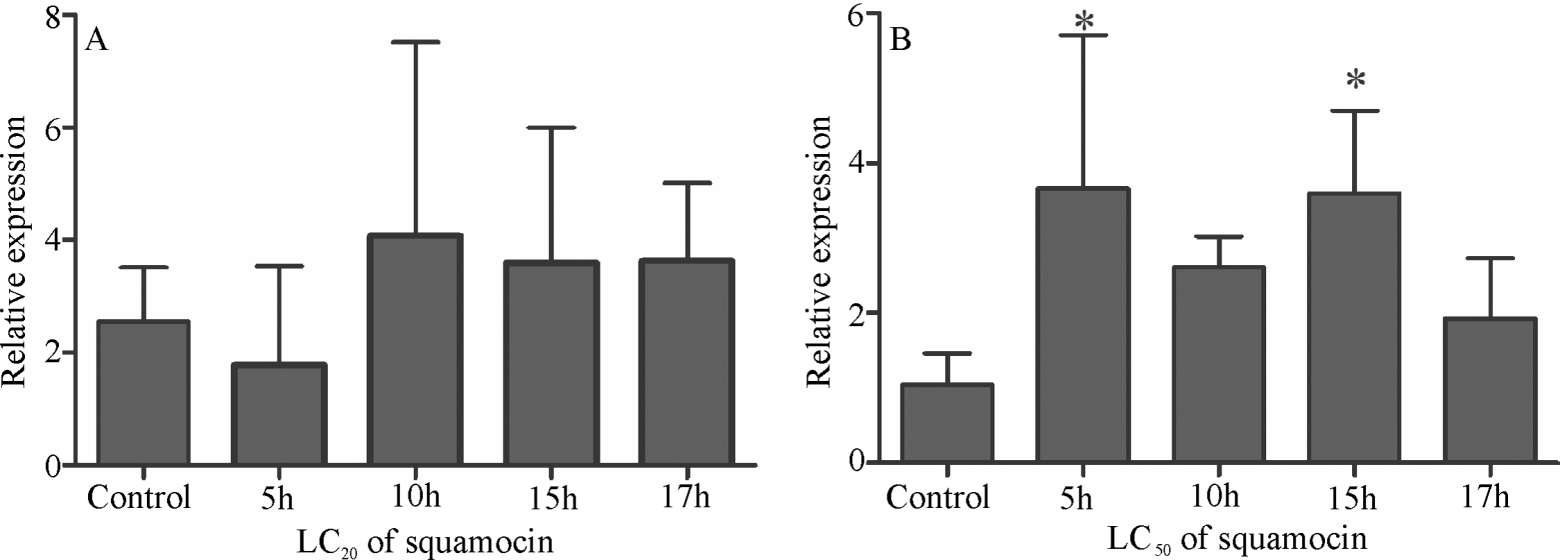
Relative mRNA levels of *Atg8* in the midgut of *Aedes aegypti* third instar larvae exposed to sublethal doses (LC_20_ and LC_50_) of squamocin and control at different times. (A) Lethal concentration of 20% of population (LC_20_). (B) Lethal concentration of 50% of population (LC_50_). The *y* axis indicates the relative gene expression corresponding to the *Atg8* mRNA levels relative to ribosomal protein rp7S (reference) gene mRNA level (mean ± se). * *p* < 0.05.

The expression of *Atg8* in the midgut of *A*. *aegypti* larvae in LC_20_ was similar to control in all exposure times (F_4,15_ = 0.7548, p = 0.5704) (Fig 5A). In the LC_50_, after 15 hours of exposure to squamocin there was higher *Atg8* expression in comparison with other periods and control (F_4,15_ = 3.900; p = 0.0230) (Fig 5B).

**Fig 6:**
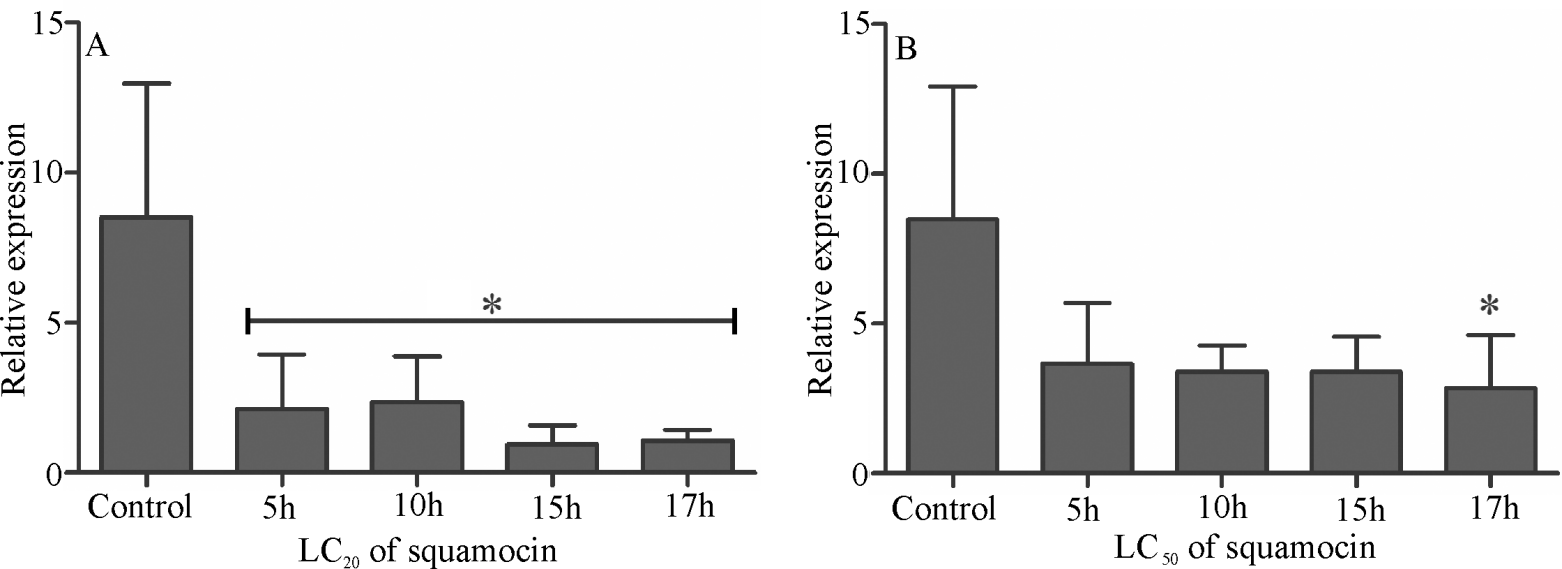
Relative mRNA levels of *V-ATPase* in the midgut of *Aedes aegypti* third instar larvae exposed to sublethal doses (LC_20_ and LC_50_) of squamocin and control at different times. (A) Lethal concentration of 20% of population (LC_20_). (B) Lethal concentration of 50% of population (LC_50_). The *y* axis indicates the relative gene expression, corresponding to the *V-ATPase* mRNA levels relative to ribosomal protein rp7S (reference) gene mRNA level (mean ± se). * *p* < 0.05.

The expression of the gene for *V-ATPase* in the midgut of *A*. *aegypti* larvae in the LC_20_ was lower than in control at all periods tested (F_4,15_ = 7.581, p = 0.0015) (Fig 6A). Larvae treated with LC_50_ for 17 hours had lower expression than other animals (F_4,15_ = 3.736, p = 0.0266) (Fig 6B).

**Fig 7:**
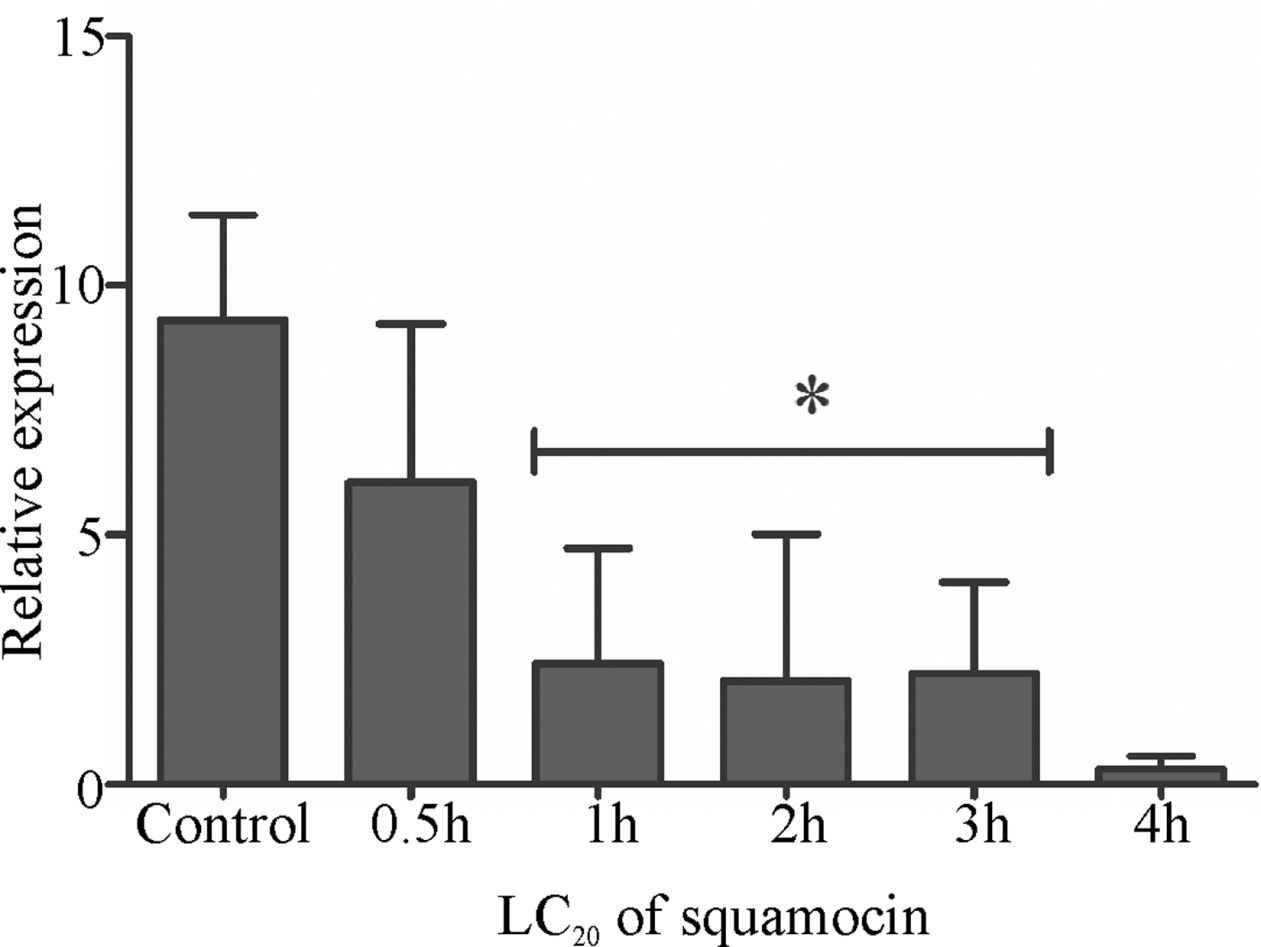
Relative mRNA levels of *Aqp4* in the midgut of *Aedes aegypti* third instar larvae exposed to sublethal dose (LC_20_) of squamocin and control at different times. The *y-axis* indicates the relative gene expression corresponding to the *Aqp4* mRNA levels relative to ribosomal protein rp7S (reference) gene mRNA level (mean ± se). * *p* < 0.05.

The expression of aquaporin gene *Aqp4* decreased when larvae were treated with LC_20_ squamocin in all periods in comparison with control (F_5,12_ = 6.204; p = 0.0046) (Fig 7). However, when subjected to LC_50_ expression was inhibited in the midgut of *A*. *aegypti* third instar larvae

## Discussion

Our results show that squamocin from *A*. *mucosa* seed affect cell morphology and physiology of *A*. *aegypti* third instar larvae in sublethal doses, and these effects may negatively affect the fitness of this vector. In the midgut digestive cells, squamocin causes loss of microvilli and intense cytoplasm vacuolization with lamellar bodies. Similar to that found for squamocin, other chemical compounds have been reported to have toxic effects against larvae of *A*. *aegypti* and other mosquitoes, with damages in the gut cells, mitochondrial cristae, brush border alterations, increase in autophagic vacuoles, membrane cell rupture, cell detachment from basal membrane, and presence of large cytoplasm vacuoles [22–27].

The presence of many vacuoles with lamellar content in the digestive cells of *A*. *aegypti* larvae treated with acetogenin, suggests a possible occurrence of cell death [25, 28]. Although the presence of vacuoles with lamellar content occurs in the midgut cells of some insects [26,27], the intense cytoplasm vacuolization has been characterized as cell death process by autophagy [29–31]. Cell death by autophagy in the digestive cells of *A*. *egypti* larvae after exposure to squamocin is supported by the increase in the *Atg1* and *Atg8* expression in these insects. There was a higher expression of *Atg1* after 1 hour of exposure to squamocin in both LC_20_ as the LC_50_, suggesting that after this short period, cells undergo autophagy. *Atg1* is a gene regulatory of autophagy induction and its is sufficient to induce hight leves of autophagy [32]. The putative complex ATG1 (serine/threonine kinase) is a protein involved in the autophagy induction [28], and its activation promotes the recruitment of other ATG proteins forming preautophagosomes [33]. The protein, ATG8, is an ubiquitin involved in the expansion of autophagosome vesicle used as a molecular marker for the autophagy [29], and the increase of *Atg8* mRNA level in the midgut of *A*. *aegypti* larvae exposed to LC_50_ squamocin indicates the occurrence of autophagy in these cells.

Neither cytotoxic effect nor changes in the *Atg8* expression are dose dependent in the midgut of *A*. *aegypti* larvae. The main function of ATG8 in the autophagy is to bind regulatory and/or autophagy-regulated proteins and guide them to their site of action in the autophagosome membrane[33,34]. This process may occur in *A*. *aegypti* larvae in the first 5 hours of squamocin exposure, or, in this period, may occur in the formation of larger autophagosome, because the amount of ATG8 determines the size of an autophagosome vacuole [35]. ATG8 is the unique structural protein known to remain in autophagosome membrane after its formation [29,35], which may explain the expression of this gene in the digestive cells of *A*. *aegypti* larvae even 17 hours after exposure to squamocin. Anyway, the presence of ATG8 occurs only after ATG1 synthesis[34–38]. Kamada *et al*. [36] supports that in the midgut digestive cells of *A*. *aegypti* larvae, squamocin promotes an increase in autophagy rate.

Changes in the brush border, and the vacuolization of apical cytoplasm of digestive cells in *A*. *aegypti* larvae exposed to sublethal doses of squamocin, may be due to changes in the water balance, as suggested for some neurotoxic insecticides in nontarget organs such as the gut [26,39–41]. The squamocin decreases the expression of *V-ATPase* gene in the midgut of *A*. *aegypti* larvae compared with the control. ATPases are cell membrane enzymes, especially in the gut epithelium, which mediate absorption and transport of metabolites and nutrients, as well as ions and nonelectrolytes [42]. In *A*. *aegypti* larvae, the V-ATPase is located in the basolateral membrane of the anterior midgut and in the apical cell membrane of the posterior midgut region [43, 44]. Thus, the decrease in the expression of *V-ATPase* may be also associated with autophagy in the midgut of larvae studied here. The relationship between autophagy and *V-ATPase* inhibition observed in *A*. *aegypti* larvae exposed to squamocin has been found in cancer cells exposed to the V-ATPase inhibitor archazolid [45].

Another effect of squamocin in the midgut in *A*. *aegypti* larvae is in the expression of aquaporin gene *Aqp4*. When treated with a low squamocin concentration (LC_20_) of the *Aqp4* expression decreased in the first hour of treatment, whereas after high concentration (LC_50_) the gene is inhibited. AQP4 is highly produced in the midgut of *A*. *aegypti*, and it is an aquagliceroporin of the aquaporin superfamily [46] playing an important role in water transport and in the absorption of polyols, urea and trehalose, which shows that AQP4 is a multifunctional membrane transporter [47]. Together, decrease and inhibition of *Aqp4* and decrease of *V-ATPase* expression in the midgut of *A*. *aegypti* larvae exposed to squamocin, suggest that this molecule may affect water and ion transport, which are important for the maintenance of cell osmotic balance, resulting in digestive cell injuries. The block of substances transported across the midgut plasma membrane may activate autophagy in these cells, which, despite its essential role for the maintenance of cell homeostasis, when activated in excess, may destroy cellular proteins and organelles, resulting in cell collapse [48]. Our findings show that squamocin increases the cytoplasm vacuolization in the digestive cells probably due to the action of *Atg8* that is increased and triggered by inhibition of *V-ATPase* and *Aqp4* genes responsible for coding two important membrane carriers.

Our study shows that squamocin in sublethal doses causes severe reactions in the midgut digestive cells of *A*. *aegypti* larvae, linked with dowregulation of *V-ATPase* and *Aqp4* genes for osmoregulatory proteins resulting in cytological abnormalities and cell death by autophagy. Thus, the squamocin from *A*. *mucosa* herein studied in sublethal doses affects physiological processes essential to the insect’s development. Generally, sublethal residues of insecticides cause effects on insect development, because for its survival it is essential that many physiological events are accurately coordinated [49].

This is the first report that an acetogenin induces autophagy, decreases *V-ATPase*, and inhibits *Aqp4* genes expression in insects. Overall, this finding is important because squamocin has multiple modes of action in the midgut of an insect larva, which may reduce the possibility of resistance of *A*. *aegypti* larvae exposed to this molecule.

## References

[1] Okwute SK. Plants as potential sources of pesticidal agents: a review. Pesticides–Advances in Chemical and Botanical Pesticides. 2012:207–32.

[2] Isman MB. Botanical insecticides, deterrents, and repellents in modern agriculture and an increasingly regulated world. Annu Rev Entomol. 2006; 51:45–66. 10.1146/annurev.ento.51.110104.151146 16332203

[3] Ahammadsahib KI, Hollingworth RM, McGovren JP, Hui YH, McLaughlin JL. Mode of action of bullatacin: a potent antitumor and pesticidal annonaceous acetogenin. Life Sci. 1993; 53(14):1113–20. 10.1016/0024-3205(93)90547-G 8371627

[4] Bermejo A, Figadère B, Zafra-Polo MC, Barrachina I, Estornell E, Cortes D. Acetogenins from Annonaceae: recent progress in isolation, synthesis and mechanisms of action. Nat Prod Rep. 2005; 22:269–03. 10.1039/B500186M 15806200

[5] Patramool S, Choumet V, Surasombatpattana P, Sabatier L, Thomas F, Thongrungkiat S, et al Update on the proteomics of major arthropod vectors of human and animal pathogens. Proteomics. 2012; 12(23–24):3510–23. 10.1002/pmic.201200300 23077092

[6] Rattan RS. Mechanism of action of insecticidal secondary metabolites of plant origin. Crop Prot. 2010; 29(9):913–20. 10.1016/j.cropro.2010.05.008

[7] Wink M. Interference of alkaloids with neuroreceptors and ion channels. Stud Nat Prod Chem. 2000; 21:3–122. 10.1016/S1572-5995(00)80004-6

[8] Halstead SB. Dengue virus-mosquito interactions. Annu Rev Entomol. 2008; 53:273–91. 10.1146/annurev.ento.53.103106.093326 17803458

[9] Ligon BL, Reemergence of an unusual disease: the chikungunya epidemic. In: Seminars in pediatric infectious diseases. 2006, 17(2): 99–104. 10.1053/j.spid.2006.04.009 16822471 PMC7128160

[10] Boorman JPT, Porterfield JS. A simple technique for infection of mosquitoes with viruses transmission of Zika virus. Trans R Soc Trop Med Hyg. 1956; 50(3):238–42. 10.1016/0035-9203(56)90029-3 13337908

[11] Regis L, Monteiro AM, Melo-Santos MAV, Silveira JC Jr, Furtado AF, Acioli RV, et al Developing new approaches for detecting and preventing *Aedes aegypti* population outbreaks: basis for surveillance, alert and control system. Mem Inst Oswaldo Cruz. 2008; 103(1):50–9. 10.1590/S0074-02762008000100008 18368236

[12] Perumalsamy H, Kim JR, Oh SM, Jung JW, Ahn YJ, Kwon HW. Novel histopathological and molecular effects of natural compound pellitorine on larval midgut epithelium and anal gills of *Aedes aegypti*. Plos one. 2013; 8(11) e80226 10.1371/journal.pone.0080226 24260359 PMC3832413

[13] Whalon M, Mota-Sanchez D, Duynslager L. The database of arthropod resistance to pesticides 2006. Available: http://www.pesticideresistance. org/DB/index. php.

[14] Luna JD, Martins MF, Anjos AF, Kuwabara EF, Navarro-Silva MA. Susceptibilidade de *Aedes aegypti* aos inseticidas temephos e cipermetrina, Brasil. Rev Saúde Públ. 2004; 38:842–3. 10.1590/S0034-89102004000600013 15608903

[15] Braga IA, Valle D. *Aedes aegypti*: inseticidas, mecanismos de ação e resistência. Epidemiol Serv Saúde. 2007; 16(4). 10.5123/S1679-49742007000400006

[16] He K, Zeng L, Ye Q, Shi G, Oberlies NH, Zhao GX, et al Comparative SAR evaluations of annonaceous acetogenins for pesticidal activity. Pest Sci. 1997; 49(4):372–8. doi: 10.1002/(SICI)1096-9063(199704)49:4<372::AID-PS543>3.0.CO; 2-K

[17] Costa MS, Pinheiro DO, Serrão JE, Pereira MJB. Morphological changes in the midgut of *Aedes aegypti* L.(Diptera: Culicidae) larvae following exposure to an *Annona coriacea* (Magnoliales: Annonaceae) extract. Neotrop Entomol. 2012; 41:311–4. 10.1007/s13744-012-0050-z 23950067

[18] Costa MS, Cossolin JFS, Pereira MJB, Sant'Ana AE, Lima MD, Zanuncio JC, et al Larvicidal and cytotoxic potential of squamocin on the midgut of *Aedes aegypti* (Diptera: Culicidae). Toxins. 2014; 6(4):1169–76. 10.3390/toxins6041169 24674934 PMC4014726

[19] Souza W. Tecnicas de microscopi eletrônica aplicada a Ciências Biológicas. 2nd ed. Rio de Janeiro: Sociedade Brasileira de Microscopia; 2007.20.

[20] Bryant B, Raikhel AS. Programmed autophagy in the fat body of *Aedes aegypti* is required to maintain egg maturation cycles. PloS one. 2011; 6(11):e25502 10.1371/journal.pone.0025502 22125592 PMC3219638

[21] Ginzinger DG. Gene quantification using real-time quantitative PCR. Exp Hematol Oncol. 2002; 30(6):503–12. 10.1016/S0301-472X(02)00806-8 12063017

[22] Cocke J, Bridges AC, Mayer RT, Olson JK. Morphological effects of insect growth regulating compounds on *Aedes aegypti* (Diptera: Culicidae) larvae. Life Sci. 1979; 24(9):817–31. 10.1016/0024-3205(79)90366-7 449622

[23] Davidson EW. Ultrastructure of midgut events in the pathogenesis of *Bacillus sphaericus* strain SSII-1 infections of *Culex pipiens quinquefasciatus* larvae. Can J Microbiol. 1979; 25(2):178–84. 10.1139/m79-028 436015

[24] Furtado RF, Lima MGA, Andrade Neto M, Bezerra JNS, Silva MGV. Atividade larvicida de óleos essenciais contra *Aedes aegypti* L. (Diptera: Culicidae). Neotrop Entomol. 2005; 34(5):843–7. 10.1590/S1519-566X2005000500018

[25] Arruda W, Cavasin GM, Silva IG. Estudo ultra-estrutural do efeito da toxicidade do extrato da *Magonia pubescens* (ST. HIL.) no mesêntero de larvas de *Aedes aegypti* (L.)(Diptera: Culicidae). Revista de Patologia Tropical. 2008; 37(3). NLM ID: 03656774.

[26] Alves SN, Serrão JE, Melo AL. Alterations in the fat body and midgut of *Culex quinquefasciatus* larvae following exposure to different insecticides. Micron. 2010; 41(6):592–7. 10.1016/j.micron.2010.04.004 20452779

[27] Fernandes KM, Gonzaga WG, Pascini TV, Miranda FR, Tomé HVV, Serrão JE, et al Imidacloprid impairs the post-embryonic development of the midgut in the yellow fever mosquito *Stegomyia aegypti* (= *Aedes aegypti*). Med Vet Entomol. 2015; 29(3):245–54. 10.1111/mve.12122 25968596

[28] Hariri M, Millane G, Guimond MP, Guay G, Dennis JW, Nabi IR. Biogenesis of multilamellar bodies via autophagy. Mol Biol Cell. 2000; 11:255–268. 10.1091/mbc.11.1.255 10637306 PMC14772

[29] Levine B, Klionsky DJ. Development by self-digestion: molecular mechanisms and biological functions of autophagy. Dev Cell. 2004; 6(4):463–77. 10.1016/S1534-5807(04)00099-1 15068787

[30] Rost-Roszkowska MM, Vilimova J, Sosinka A, Skudlik J, Franzetti E. The role of autophagy in the midgut epithelium of *Eubranchipus grubii* (Crustacea, Branchiopoda, Anostraca). Arthropod Struct Dev. 2012; 41(3):271–9. 10.1016/j.asd.2012.01.001 22445350

[31] Santos DE, Azevedo DO, Campos LAO, Zanuncio JC, Serrão JE. *Melipona quadrifasciata* (Hymenoptera: Apidae) fat body persists through metamorphosis with a few apoptotic cells and an increased autophagy. Protoplasma. 2015; 252(2):619–27. 10.1007/s00709-014-0707-z 25269629

[32] Levine B, Klionsky D. Development by self-digestion: molecular mechanisms and biological functions of autophagy. Dev Cell. 2004; 6:463–477. 10.1016/S1534-5807(04)00099-1 15068787

[33] Ichimura Y, Kirisako T, Takao T, Satomi Y, Shimonishi Y, Ishihara N, et al A ubiquitin-like system mediates protein lipidation. Nature. 2000; 408(6811):488–92. 10.1038/35044114 11100732

[34] Hu C, Zhang X, Teng YB, Hu HX, Li WF. Structure of autophagy-related protein Atg8 from the silkworm *Bombyx mori*. Acta Crystallogr F Struct Biol Commun. 2010; 66(7):787–90. 10.1107/S1744309110018464 PMC2898461 20606273

[35] Klionsky DJ, Schulman BA. Dynamic regulation of macroautophagy by distinctive ubiquitin-like proteins. Nat Struct Mol Biol. 2014; 21(4):336–45. 10.1038/nsmb.2787 24699082 PMC4036234

[36] Kamada Y, Yoshino K, Kondo C, Kawamata T, Oshiro N, Yonezawa K, et al Tor directly controls the Atg1 kinase complex to regulate autophagy. Mol Cell Biol. 2010; 30(4):1049–58. 10.1128/MCB.01344-09 19995911 PMC2815578

[37] Cheong H, Nair U, Geng J, Klionsky DJ. The Atg1 kinase complex is involved in the regulation of protein recruitment to initiate sequestering vesicle formation for nonspecific autophagy in *Saccharomyces cerevisiae*. Mol Biol Cell. 2008; 19(2):668–81. 10.1091/mbc.E07-08-0826 18077553 PMC2230592

[38] Nakatogawa H, Ohbayashi S, Sakoh-Nakatogawa M, Kakuta S, Suzuki SW, Kirisako H, et al The autophagy-related protein kinase Atg1 interacts with the ubiquitin-like protein Atg8 via the Atg8 family interacting motif to facilitate autophagosome formation. J Biol Chem. 2012; 287(34):28503–7. 10.1074/jbc.C112.387514 22778255 PMC3436563

[39] Cooper SG, Douches DS, Zarka K, Grafius EJ. Enhanced resistance to control potato tuberworm by combining engineered resistance, avidin, and natural resistance derived from, *Solanum chacoense*. Am J Potato Res. 2008; 86(1):24–30. 10.1007/s12230-008-9057-8

[40] Correia A, Wanderley-Teixeira V, Teixeira ÁAC, Oliveira JV, Torres JB. Morfologia do canal alimentar de lagartas de *Spodoptera frugiperda* (JE Smith) (Lepidoptera: Noctuidae) alimentadas com folhas tratadas com nim. Neotrop Entomol. 2009; 38(1):083–91. 10.1590/S1519-566X2009000100008 19347100

[41] Almeida GD, Zanuncio JC, Senthil-Nathan S, Pratissoli D, Polanczyk RA, Azevedo DO, et al Cytotoxicity in the midgut and fat body of *Anticarsia gemmatalis* (Lepidoptera: Geometridae) larvae exerted by neem seeds extract. Invertebrate Surviv J. 2014; 11:79–86.

[42] Wieczorek H, Grüber G, Harvey WR, Huss M, Merzendorfer H. The plasma membrane H+-V-ATPase from tobacco hornworm midgut. J Bioenerg Biomembr. 1999; 31(1):67–74. 10.1023/A:1005448614450 10340850

[43] Zhuang Z, Linser PJ, Harvey WR. Antibody to H (+) V-ATPase subunit E colocalizes with portasomes in alkaline larval midgut of a freshwater mosquito (*Aedes aegypti*). J Exp Biol. 1999; 202(18):2449–60. PMID:. 10460732 10.1242/jeb.202.18.2449

[44] Patrick ML, Aimanova K, Sanders HR, Gill SS. P-type Na+/K+-ATPase and V-type H+-ATPase expression patterns in the osmoregulatory organs of larval and adult mosquito *Aedes aegypti*. J Exp Biol. 2006; 209(23):4638–51. 10.1242/jeb.02551 17114398

[45] Schwarzenberg K, Wiedmann RM, Oak P, Schulz S, Zischka H, Wanner G, et al Mode of cell death induction by pharmacological vacuolar H+-ATPase (V-ATPase) inhibition. J Biol Chem. 2013; 288(2):1385–96. 10.1074/jbc.M112.412007 23168408 PMC3543021

[46] Finn RN, Chauvigné F, Stavang JA, Belles X, Cerdà J. Insect glycerol transporters evolved by functional co-option and gene replacement. Nat Commun. 2015; 6 10.1038/ncomms8814 PMC4518291 26183829

[47] Drake LL, Rodriguez SD, Hansen IA. Functional characterization of aquaporins and aquaglyceroporins of the yellow fever mosquito, *Aedes aegypti*. Sci Rep. 2015; 5 10.1038/srep07795 PMC4295104 25589229

[48] Mizushima N, Levine B, Cuervo AM, Klionsky DJ. Autophagy fights disease through cellular self-digestion. Nature. 2008; 451(7182):1069–75. 10.1038/nature06639 18305538 PMC2670399

[49] Desneux N, Decourtye A, Delpuech JM. The sublethal effects of pesticides on beneficial arthropods. Annu Rev Entomol. 2007; 52:81–106. 10.1146/annurev.ento.52.110405.091440 16842032

